# *Pseudomonas aeruginosa* clinical isolates in Egypt: phenotypic, genotypic, and antibiofilm assessment of Pluronic F-127

**DOI:** 10.1186/s12866-025-03946-0

**Published:** 2025-04-25

**Authors:** Mai Hamed Salem, Ahmed F. Azmy, Tarek Dishisha, Nesrein Dessouky

**Affiliations:** 1 Department of Microbiology and Immunology, College of Pharmaceutical Sciences and Drug Manufacturing, Misr University for Sciences and Technology, P.O. Box 77, Giza, Egypt; 2 Department of Pharmaceutical Microbiology and Immunology, Faculty of Pharmacy, Beni-Suef University, Beni-Suef, 62511 Egypt

**Keywords:** *Pseudomonas aeruginosa*, Antibiotic resistance, Quorum sensing, Biofilm, Pluronic F-127

## Abstract

**Background:**

Virulence factors play an important role in developing bacterial resistance leading to the increased severity of *Pseudomonas aeruginosa* infections. Several genes encoding for virulence factors is coordinated by the quorum sensing (QS) system. In the present study, the prevalence of virulence genes, particularly those involved in controlling biofilm formation, and their correlation with antibiotic resistance patterns was investigated. The ability of the pathogens to form biofilm and the impact of Pluronic F-127 as a potential biofilm inhibitor was assessed.

**Results:**

A total of 118 *P. aeruginosa* clinical isolates were collected. The highest resistance rates were observed against ceftazidime (94%), while colistin was the most effective followed by polymyxin B with sensitivity rate 72% and 59%, respectively. Out of 118 isolates: 111 (94%) were biofilm producers, 24.6% of them were strong. The QS genes; *las*R and *rhl*R, were detected in 85% and 89% of the isolates, respectively, *toxA* gene in 95% and *ampC* gene in 69% of the isolates. Pluronic F-127 was confirmed as a biofilm inhibitor in lowest concentration used 1.25 mg/ml which inhibits 78% of strong biofilm forming isolates and has better effect on detachment of established biofilm by 90% of biofilm forming isolates.

**Conclusion:**

The ability of bacteria to form biofilms contributes greatly to the development of antibiotic resistance, which leads to the occurrence of persistent and chronic bacterial illnesses. Many isolates exhibited moderate to strong biofilm forming ability, which showed a high resistance pattern. The results demonstrated that Pluronic F-127 has a promising level of biofilm inhibition and detachment in most isolates. It has a chance to serve as a substitute means for combating biofilm formation.

**Supplementary Information:**

The online version contains supplementary material available at 10.1186/s12866-025-03946-0.

## Background

*Pseudomonas aeruginosa* is a Gram-negative, widely spread bacteria. It is an opportunistic pathogen causing both chronic and life-threatening infections; notably in hospitalized and immunocompromised patients [1]. It is responsible for several infectious diseases as meningitis, endocarditis, ventilator-associated pneumonia (VAP), urinary tract infections, endophthalmitis and external otitis [2]. Patients with burns, chronic obstructive pulmonary disease (COPD), cancer, traumas, and cystic fibrosis (CF) are the most prone to these infections. Therefore, *P. aeruginosa* is one of the top-listed pathogens leading to nosocomial infections, frequently associated with medical devices such as ventilators and catheters and tends to develop well on wet surfaces [3].

The resistance of *P. aeruginosa* to antimicrobial agents has become a worldwide problem, especially with the growing events of multi- (MDR) and extensive- (XDR) drug resistant strains [4], attracting more attention for its resistance pattern, virulence factors, and physiological adaptability. *P. aeruginosa* exhibits inherent resistance to certain antimicrobials through various mechanisms, primarily due to the pathogen’s remarkable capacity to form biofilms [5]. These biofilms consist of bacterial communities that adhere to surfaces and proliferate under the protection of an exopolysaccharide matrix (EPS), making the treatment of *P. aeruginosa* infections more challenging [6]. The production of biofilm is a sequential process including microbial surface attachment, cell multiplication, matrix synthesis, and dissociation [7]. According to the Center for Disease Control and Prevention (CDC), biofilms are responsible for more than 65% of illnesses in developed countries [8]. Implanted devices such as catheters, orthopedic prostheses, and heart valves are susceptible to infection, as frequent as conditions like gingivitis and middle ear infections in children. Probably the most well-known biofilm-producing organism is the *P. aeruginosa* which infects cystic fibrosis patients causing a highly persistent infection [9].

Biofilm Formation has been regulated by bacterial cell to cell communication mechanism known as quorum sensing (QS) [10]. QS is crucial for orchestrating the synchronized gene expression of bacteria and their biological functions. Once the density of environmental microbes hits a specific threshold, the amount of signaling molecules rises, and these chemical signals are sent by receptor proteins to reach the inside of cells, thereby modifying the expression of specific genes, and regulating physiological processes [11]. Three QS systems occur in *P. aeruginosa*, two of these are LuxI/LuxR-type QS circuits that work in series to regulate the production of virulence proteins. The third one is a non-LuxI/LuxR-type QS circuit which is known as Pseudomonas quinolone signal (PQS) that is tightly allied to the LasI/LasR and RhlI/RhlR QS systems and, as a result, affects the production of virulence factors [12].

Two autoinducer (AIs) signaling molecules connect QS circuits and virulence genes. The acyl-homoserine lactone (AHL) which is synthesized by the *las*I gene’s synthase and is detected by the *las*R gene by the transcriptional regulator, the *las* and *rhl* genes regulate the signaling network [13]. The expression of numerous genes, including those that have virulence factors and quorum sensing, was stimulated by the *las*R-AHL complex [14]. The second N-butanoyl-L-homoserine lactone (C_4_HSL) signal is produced by the same mechanism for the *rhlI* gene that was identified by the transcriptional regulator gene *rhl*R. When *rhl*R-C_4_HSL complex binds to specific locations, the virulence genes and other components are expressed [15].

To effectively control biofilm infections, an innovative strategy needs to be developed. The use of antibiofilm chemicals is interesting because it can reduce the current transmission by reducing colonization and attachment, or it can be used in conjunction with other antibiotics to increase their activity [16]. A variety of substances have been employed as antibiofilm agents including quorum sensing inhibitors such as N-acetyl homoserine, antimicrobial peptides, enzymes, and sub-MIC concentrations of antibiotics [17]. Pluronic F-127, also known as Poloxamer 407, is a copolymer composed of poly- (ethylene oxide) (PEO) and poly- (propylene oxide) (PPO) units. It belongs to a class of non-ionic surfactants and is commonly used in the pharmaceutical industry [18]. Pluronic F-127 is known for its amphiphilic nature, meaning it has both hydrophilic and hydrophobic components which gives it a good surfactant property [19].

Understanding the mechanisms of Pluronic F-127 can provide insights into its application in biomedical fields [20]. Pluronic F-127’s hydrophobic and hydrophilic characteristics contribute significantly to its capacity to disrupt biofilms. The polymer forms a hydrated coating on surfaces, reducing bacterial adherence. The process is a steric barrier, in which the hydrophilic PEO chains resist approaching bacteria, inhibiting adhesion and subsequently biofilm formation [21].

Biofilms are mostly made up of EPS, which contains polysaccharides, proteins, and nucleic acids that aid in bacterial aggregation and stability. Pluronic F-127 has been found to disturb the structural integrity of biofilms by decreasing the generation of essential EPS components, this can result in thinner biofilms that are more susceptible to environmental pressures [22].

The present study aims to evaluate the antibiotic resistance pattern, biofilm formation capability, other virulence factors contributed to the pathogenicity of *P. aeruginosa* isolated from Egyptian Hospitals. Moreover, the ability of Pluronic F-127 to inhibit biofilm formation and detach the already established biofilm as a new tactic to inhibit biofilm formation in clinical isolates investigated.

## Methods

### Chemicals and culture media

Nutrient agar, cetrimide agar and tryptone were purchased from Difco Laboratories (USA), while tryptone soya broth and yeast extract were from Oxoid Chemical co. (England). Muller-Hinton broth and bacteriological agar were products of Himedia Ltd. (India), and oxidase reagent was procured from BioMerieux (France), crystal violet stain from Oxford (India), glucose and sodium chloride from El-Nasr Chemical Co. (Egypt), and acetic acid from Aldrich Chemical Co. Ltd. (England).

### Sample collection

A total of 210 non-duplicated Gram-negative bacilli were recovered from inpatients with clinically diagnosed skin/soft tissue infections, respiratory tract infections, urinary tract infections, wound infections, bacteremia, bacterial peritonitis and meningitis from four different Egyptian hospitals (El Mansoura University Hospital, El Demerdash Hospital, Atfal-Misr Hospital and the Memorial Souad-Kafafy University Hospital) over the period from November 2021 until March 2022. The study was conducted using clinical specimens collected from cases that developed their infections 48 h after hospital admission. Samples were promptly transported to the laboratory and examined microscopically using the Gram-staining technique.

### Isolation, identification and storage of isolates

Collected samples in the previous step were transferred to MacConkey agar as selective and differential media, and cetrimide agar as selective media for *P. aeruginosa* and the plates were incubated at 37 °C for 24 h. The isolates were further tested microscopically and biochemically, positive oxidase and catalase. Genotypic confirmation of the isolates was done by *toxA* gene [23]. The different isolates were preserved as glycerol stocks (20% v/v) at − 80 °C. Growing bacteria on fresh plates from frozen stocks is critical when starting experiments [24].

### Antibiotic susceptibility test

The standard Kirby-Bauer disc-diffusion method was employed for evaluating the antibiotic susceptibility of *P. aeruginosa* isolates to different antibiotic classes. The test was carried out according to the Clinical and Laboratory Standards Institute (CLSI) guidelines using gentamicin (CN, 10 mg), tobramycin (TOB, 10 mg), amikacin (AK, 30 mg), streptomycin (S, 10 mg), piperacillin/tazobactam (TPZ, 110 mg), ciprofloxacin (CIP, 5 mg), levofloxacin (LEV, 5 mg), meropenem (MEM, 10 mg), ceftriaxone (CRO, 30 mg), amoxicillin/clavulanic acid (AMC, 30 mg), ceftazidime (CAZ, 30 mg), imipenem (IPM, 10 mg), colistin(CLM, 10 mg), and polymyxin B (PB, 300 mg) discs. Results were recorded for each isolate and categorized as sensitive, multi- (MDR), extensive- (XDR) or pan- drug resistant (PDR) [25].

### Determination of colistin resistance via the minimum inhibitory concentration (MIC)

The MIC of colistin was also determined using broth microdilution technique according to CLSI recommendations. The range of colistin concentrations utilized was 0.25 to 8 µg/ml. As a positive control of bacterial growth, Mueller-Hinton broth inoculated with *P. aeruginosa* without any antibiotic treatment was utilized. The MIC was identified as the lowest concentration at which the bacterial growth was clearly inhibited [26].

### Biofilm formation assay

The biofilm formation was evaluated by microtiter plate method (MTPM) using 96-well flat-bottomed tissue culture plates [27]. Initially, 10 µl of the stock solution of the isolates was streaked to the surface of tryptone soya agar medium, which was incubated at 37 °C for 24 h. The resulting growth was transferred to 10 ml trypticase soya broth (TSB) media supplemented with 1% filter-sterilized glucose and left at 37 °C for 24 h. Bacterial suspension was then diluted 1:100 with fresh TSB, and 200 µl was transferred to the microtiter plate wells, the negative controls well contained only sterile broth. The plates were incubated statically at 37 °C for 24 h. Subsequently, the free unbound planktonic cells were discarded, and the wells were washed three times using bi-distilled water and then left to dry. The formed biofilm was fixed by incubation at 60 °C for 30 min in an oven. The attached cells in each well were then stained by 250 µl of 1%(w/v) crystal violet (CV) for 20 min. The stain was discarded, and the plates were immersed in bi-distilled water three times to remove the excess stain, then left upside down to dry. The cell-bound stain was solubilized in 250 µl of 30% acetic acid and left for 30 min. The optical density (OD) was measured at 595 nm using a microplate ELISA reader (BioTek^®^800™). The experiment was performed in six independent replicates.

The interpretation of results was performed according to the criteria of Stepanovic et al. (2007) [28]. Briefly, the Optical Density Cutoff (OD_C_); a threshold value derived from the optical density (OD) measurements obtained during a biofilm quantification assay, is calculated as follows:

*OD*_*C*_ = Average OD of Negative Control + (3 × Standard Deviation of Negative Control).

Accordingly, the microorganisms are classified to:

- Non-biofilm producer: when *OD* ≤ *OD*_*C*_.
- Weak biofilm producer: when *OD*_*C*_ < *OD* ≤ 2 × *OD*_*C*_.
- Moderate biofilm producer: when 2 × *OD*_*C*_ < *OD* ≤ 4 × *OD*_*C*_.
- Strong biofilm producer: when *OD* > 4 × *OD*_*C*_.

### Swarming motility assay

Swarming motility was carried out using swarm plates formulated by nutrient agar (0.5% agar) and supplemented with 0.5% (w/v) filter-sterilized glucose. Isolates were point inoculated using sterile toothpick and left inside the incubator at 30 °C for 18 h [29].

### Swimming motility assay

Swimming motility was assessed using tryptone swim plates prepared as follows (w/v); 1% tryptone, 0.5% NaCl, and 0.3% agar. Isolates were inoculated using sterile toothpick in the middle of the plate which was then incubated at 25 °C for 18 h. Results were assessed by examining the circular zone of the growth produced as the bacterial cells moving away from the inoculation site [30].

### Detection of virulence genes using polymerase chain reaction (PCR)

The DNA was extracted from the different isolates using heat lysis method [31]. Amplification of the desired genes was carried out using Thermocycler (Bio-Rad^®^ C-1000, USA) with a total reaction volume of 20 µl containing: 10 µl PCR RedTaq^®^ Master Mix, 1 µl of each primer (Table 1), 200–400 ng of DNA and the volume was completed using nuclease-free water. The cycling condition was adjusted as follows: an initial denaturation at 95 °C for 5 min, followed by 25 cycles of denaturation at 95 °C for 20 s, then the annealing step for 30 s, and extension at 72 °C for 1 min. This was followed by a final extension step at 72 °C for 10 min [32]. The PCR products were separated by agarose gel electrophoresis using 0.8% agarose and Tris-Acetate EDTA (TAE) containing 0.3 µl of ethidium bromide as a running buffer. The separated bands were visualized under UV light.

**Table 1:** List of primers used in the genomic and transcriptomic analysis of *Pseudomonas aeruginosa* clinical isolates

Reaction	Gene	Sequence (5’ − 3’)	Annealing temp. (°C)	Amplicon size (bp)	Ref
Genomic, B-lactamase	*ampC*	F: GACAACGCCCTCAGCATCACCAG R: CGCTGGCCCATTCGCTCCAGCGCT	59	396	[33]
Genomic, Endotoxin A	*toxA*	F: GGTAGTTGGTCGCTGAAC R: GACGAAGAAGGTGGCATC	55	177	[23]
Genomic, QS regulator	*rhlR*	F: CAATGAGGAATGACGGAGGC R: GCTTCAGATGAGGCCCAGC	55	730	[34]
Genomic, QS regulator	*lasR*	F: ATGGCCTTGGTTGACGGTT R: GCAAGATCAGAGAGTAATAAGACCCA	55	725	[34]
Transcriptomic QS regulator	*lasR*	F: ACGCTCAAGTGGAAAATTGG R: TCGTAGTCCTGGCTGTCCTT	111	58	[35]
Transcriptomic QS regulator	*rhlR*	F: CATCCGATGCTGATGTCCAACC R: ATGATGGCGATTTCCCCGGAAC	101	60	[35]
Transcriptomic House keeping	*16 S rRNA*	F: TGGCTGTCGTCAGCTCGTGT R: GTCATCCCCACCTTCCTCCG	136	62	[36]

### Effect of pluronic F-127 on biofilm formation process

An overnight culture of the isolates was diluted in TSB supplemented with 1% glucose to a final concentration of 10^8^ CFU/ml in a flat bottom microtiter plate [16]. Different concentrations of Pluronic F-127 (1.25, 2.5 and 5 mg/ml) were added representing sub-MIC. The plates were incubated at 37 °C for 48 h. Extent of biofilm inhibition was detected by CV method. The percentage of biofilm inhibition was calculated by the following equation [37].

$$\displaylines{ Inhibition\,\% = \cr 100 - \left( {{{OD\,sample} \mathord{\left/{\vphantom {{OD\,sample} {OD\,control}}} \right.\kern-\nulldelimiterspace} {OD\,control}}} \right) \times 100 \cr} $$

### Effect of pluronic F-127 on pre-formed biofilm adherence

For evaluating the ability of Pluronic F-127 to breakdown the established biofilm, initially, 80 µl of inoculum was applied to a polystyrene plate followed by 120 µl of TSB and the plates were incubated for 24 h at 37 °C to allow biofilm formation. The supernatant was carefully removed under aseptic conditions and then 80 µl of 10 mg/ml Pluronic F-127 solution (water in case of negative control) were added to each well, along with 120 µl of TSB. The crystal violet staining experiment was used to determine the eradication ability following a 24-hour incubation period. Optical densities were measured and compared to the control. Every test was run in four independent replicates [38].

### Evaluating biofilm Inhibition using scanning Electron microscope (SEM)

A glass cover slip was placed in a 6-well microtiter plate containing TSB and the bacterial strain, with and without Pluronic F-127 (5 mg/ml). The plate was incubated at 37 °C for 48 h. The formed biofilm on the coverslips was fixed overnight at 4 °C using 4% glutaraldehyde in phosphate buffer saline (pH 7.4). The dehydration step was done with gradual concentrations of ethanol 50, 60, 70, 80, 90, and 100% (v/v), respectively, each for 15 min, and then allowed to dry, and subsequently coated with gold using gold sputter [39]. Bacterial biofilm was examined in a 5000 LV JEOL Scanning Electron Microscope (JEOL Ltd., Hertfordshire, UK).

### Effect of Pluronic F-127 on expression of *las*R and *rhl*R genes

*las*R and *rhl*R genes are the most reported biofilm-forming genes in *P. aeruginosa* strains. Quantitative reverse-transcriptase PCR (qRT-PCR) was employed to quantify the expression of these genes, using the 16 S rRNA house-keeping gene as a reference for comparison (Table 1). The measurement of gene expression of the two genes in *P. aeruginosa* isolates was done before and after treatment with Pluronic F-127.

Bacterial RNA was extracted using the Quantitect SYBR green PCR kit (Qiagen, Germany) in accordance with the instructions of the RNeasy Mini Kit (QIAGEN, Germany). Briefly, bacterial cells were lysed using a lysis buffer, followed by ethanol addition to facilitate RNA binding. The lysate was transferred to a RNeasy spin column, and RNA was captured on the membrane. Washing steps with washing buffers were performed to remove contaminants, and RNA was eluted with RNase-free water. RNA samples were analyzed by the NanoDrop (Nano-Drop One, Thermo Fisher, USA) [40]. The reaction volume for the RT-PCR was 25 µl as follows: 2X QuantiTect SYBR Green PCR Master Mix (12.5 µl), reverse transcriptase (0.25 µl), 0.5 µl of forward and reverse primers, 8.25 µl of RNase free water, and 3 µl of template RNA. The *Ct* of each sample was compared with the control group’s *Ct* using the “*∆∆Ct*” method to assess the variance of gene expression on the RNA of the different samples [41].

*Whereas*:

$$\Delta \Delta Ct = \Delta Ct\,reference - \Delta Ct\,t\arg et$$

$$\displaylines{\Delta Ct\,t\arg et = \cr Ct\,control - Ct\,treatment\,and\,\Delta Ct\,reference = \cr Ct\,control - Ct\,treatment \cr} $$

### Statistical analysis

Statistical analysis was performed using GraphPad Prism (version 23). One-way ANOVA test was used to evaluate significance of the difference between groups. The differences were considered statistically significant at *p*-values < 0.05.

## Results

### Isolation and identification of isolates

**Fig. 1:**
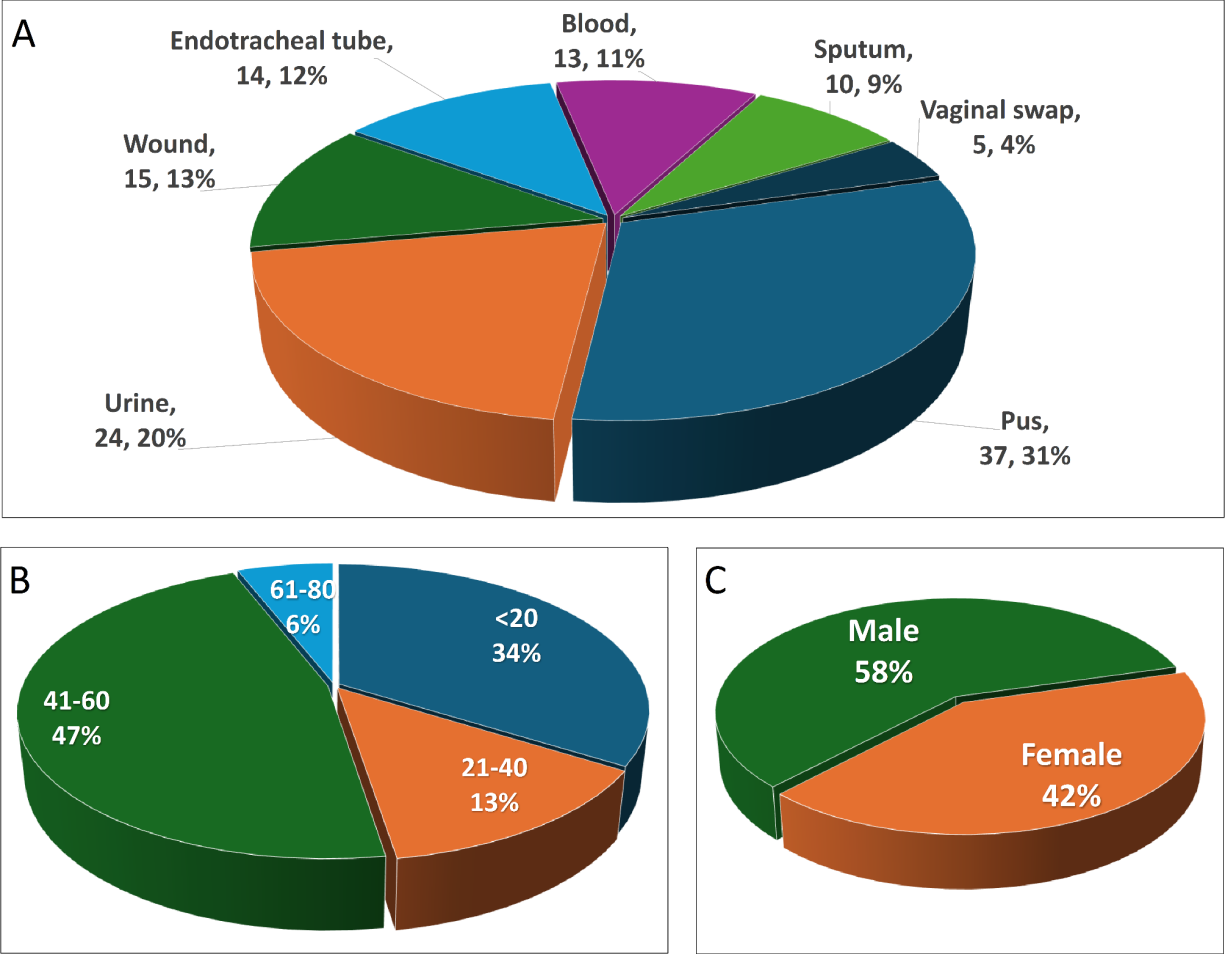
Demographic data of the different *P. aeruginosa* clinical isolates, (**A**) According to the source of the sample, showing the number of isolates obtained from each source and the percentage, (**B**) according to age group, and (**C**) according to sex

Out of the 210 clinical isolates recognized as Gram-negative bacilli, 118 isolates were identified as *P. aeruginosa* based on morphological examination, Gram-stain, and the ability to grow on the selective media. The highest number of *P. aeruginosa* was collected from pus followed by urine samples, wound infections, and endotracheal tubes (90/118). The residual isolates were collected from blood samples, sputum, and vaginal infections (28/118) (Fig. 1). The detailed demographic data of collected strains are summarized in Fig. 1 and Table S1.

### Antibiotic susceptibility testing

**Fig. 2:**
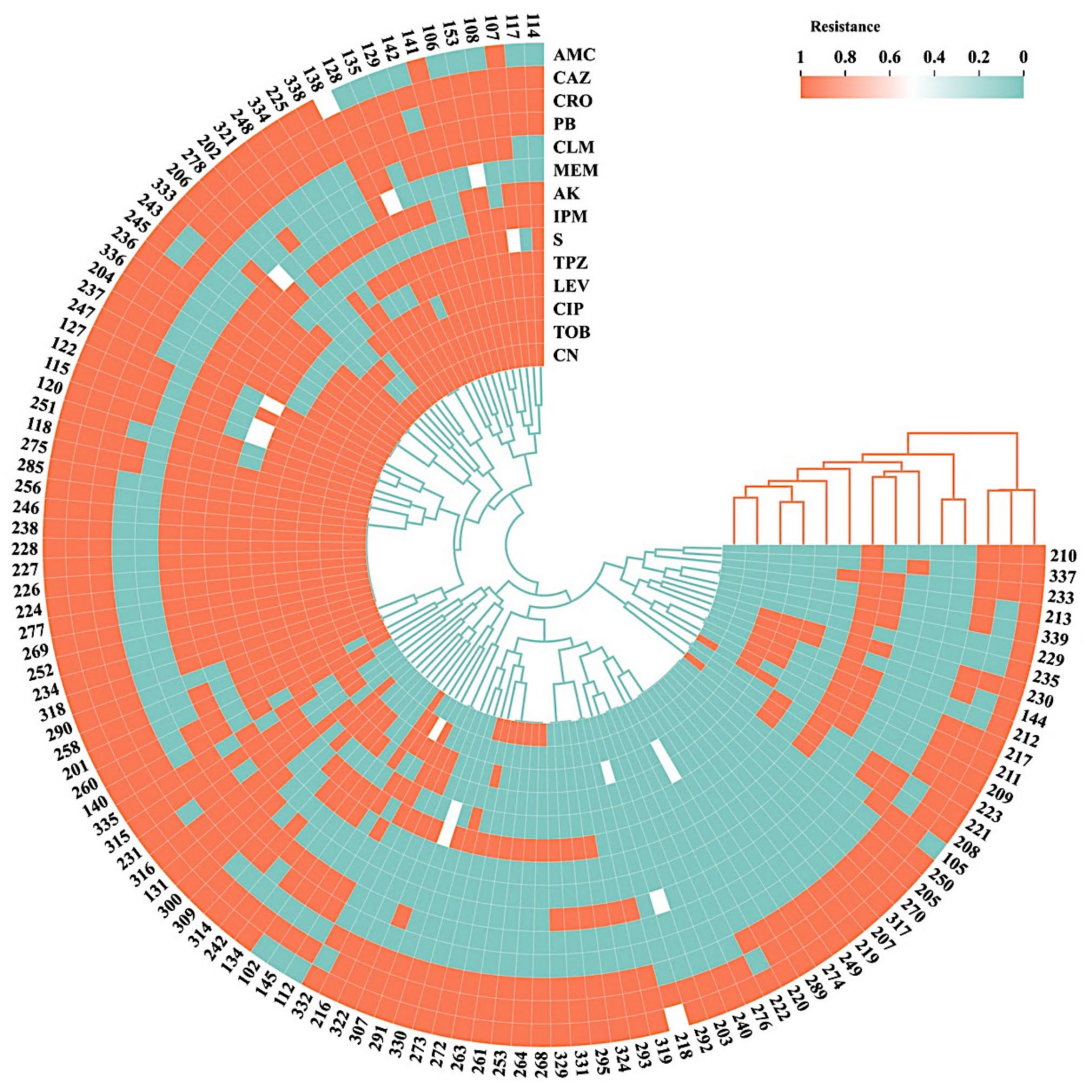
Heatmap showing the resistance pattern of the different *P. aeruginosa* clinical isolates to 14 antibiotics representing 6 different antibiotic classes where resistance is shown in orange, intermediate in white, and sensitive in green. The antibiotics (*x*-axis) and *P. aeruginosa* strains (*y*-axis) are clustered using the Euclidean clustering function based on proximity. The plot was generated using ChiPlot (Chiplot.online)

The susceptibility of the different isolates was determined to 14 antibiotics representing 6 different antibiotic classes including aminoglycosides (4 members), carbapenems (2 members), fluoroquinolones (2 members), penicillin/B-lactamase inhibitor (2 members), polymyxin (2 members) and 3rd generation cephalosporins (2 members). The resistance pattern of each of the 118 isolates is presented in Fig. 2. Overall, most of the isolates were resistant to ceftazidime (92%), ampicillin/clavulanic acid (87%), and ceftriaxone (84%), while almost 50% of the isolates showed good sensitivity for tobramycin, amikacin, piperacillin/tazobactam, meropenem and imipenem. Polymyxins were the most active group of antibiotics showing susceptibility rates of 72% and 59% of colistin and polymyxin B, respectively. Based on this pattern, *P. aeruginosa* isolates were classified as either MDR (70% of the isolates) and XDR (30% of the isolates), while no PDR pattern was observed.

### Determination of MIC of colistin

The MIC values of colistin to the different isolates were determined separately using the broth microdilution technique. The results ranged between 0.5 and 8 µg/ml. The highest proportion of the isolates were sensitive to ≤2 µg/ml (90 isolates), while 25 and 3 isolates resist 4 and 8 µg/ml colistin, respectively. This indicated that 76.2% of the isolates were sensitive to colistin, 21.3% showed intermediate resistance, while only 2.5% were resistant to 8 µg/ml colistin.

### Assessment of biofilm formation

Out of 118 isolates; 111 (94.06%) were biofilm producers; 29 isolates (24.6%) were classified as strong biofilm producers, 46 isolates (39%) were considered moderate biofilm producers, and 36 isolates (30.51%) were weak biofilm producers, whereas only 7 (5.93%) isolates didn’t produce any measurable biofilm.

### Assessment of swarming and swimming motility

All isolates were assayed for swarming and swimming motility. Ninety-one isolates (77.12%) showed positive swarming, and 109 isolates (92.37%) showed positive swimming, while only 4 isolates were negative for both tests. Eighty-six isolates (72.8%) showed positive for both swarming and swimming motilities while the residual 28 isolates showed only one of them.

### Polymerase chain reaction

**Fig. 3:**
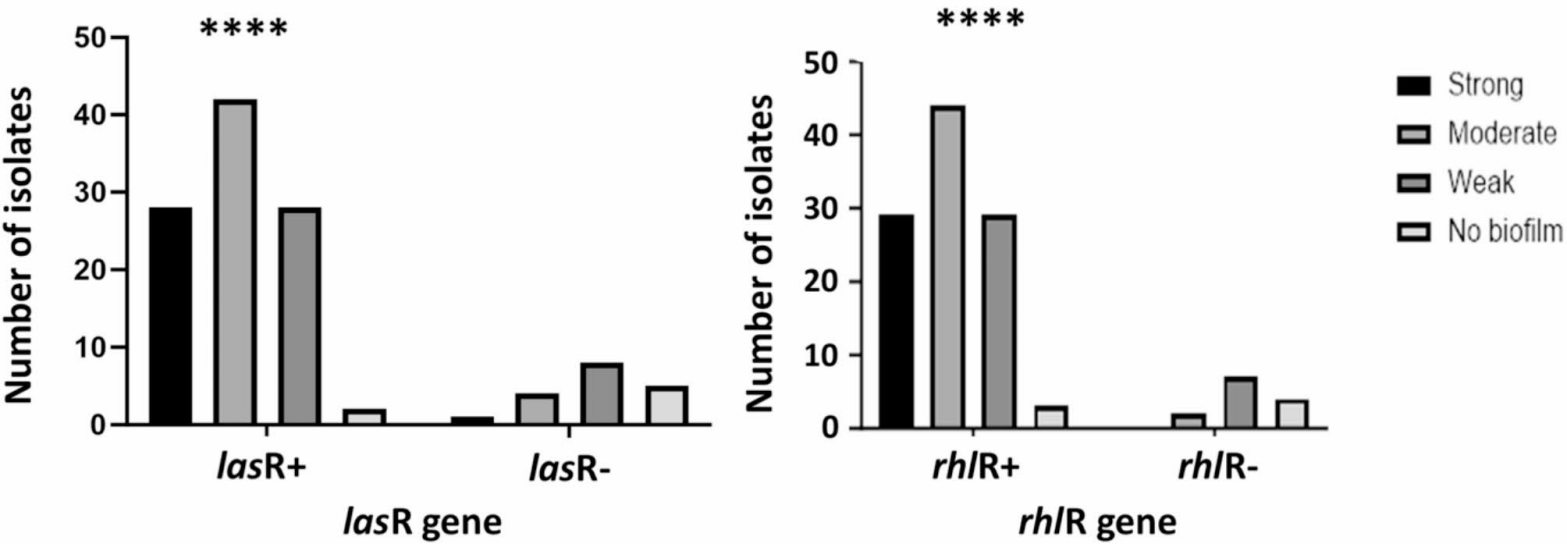
Biofilm formation among *P. aeruginosa* isolates in relation to the presence or absence of *lasR* and *rhlR* genes. The bar charts represent the number of isolates categorized based on their biofilm formation capacity on X-axis and the genes illustrated on Y-axis, Statistical significance is denoted by **** (*p* < 0.0001)

Images of the agarose gel showing a number of bands of the PCR products for the different isolates are shown in Fig. S1. Virulent *toxA* gene was detected in 95% (*n* = 112) of isolates. Results of PCR analysis of *ampC* resistance gene in *P. aeruginosa* were 69% (*n* = 81) *ampC*^+^ isolates. The results of the PCR amplification of biofilm regulating genes, *las*R and *rhl*R, showed a reasonable outcome where most of the biofilm producers were positive to *lasR* and *rhlR* genes. While non-biofilm producers were mostly negative to these genes. In *rhl*R gene, it was detected in 105 (89%) out of 118 *P. aeruginosa* isolates, while *las*R gene was detected in 100 (85%) out of 118 *P. aeruginosa* isolates (Fig. 3).

### Effect of pluronic F-127 on biofilm formation and adherence Inhibition

**Fig. 4:**
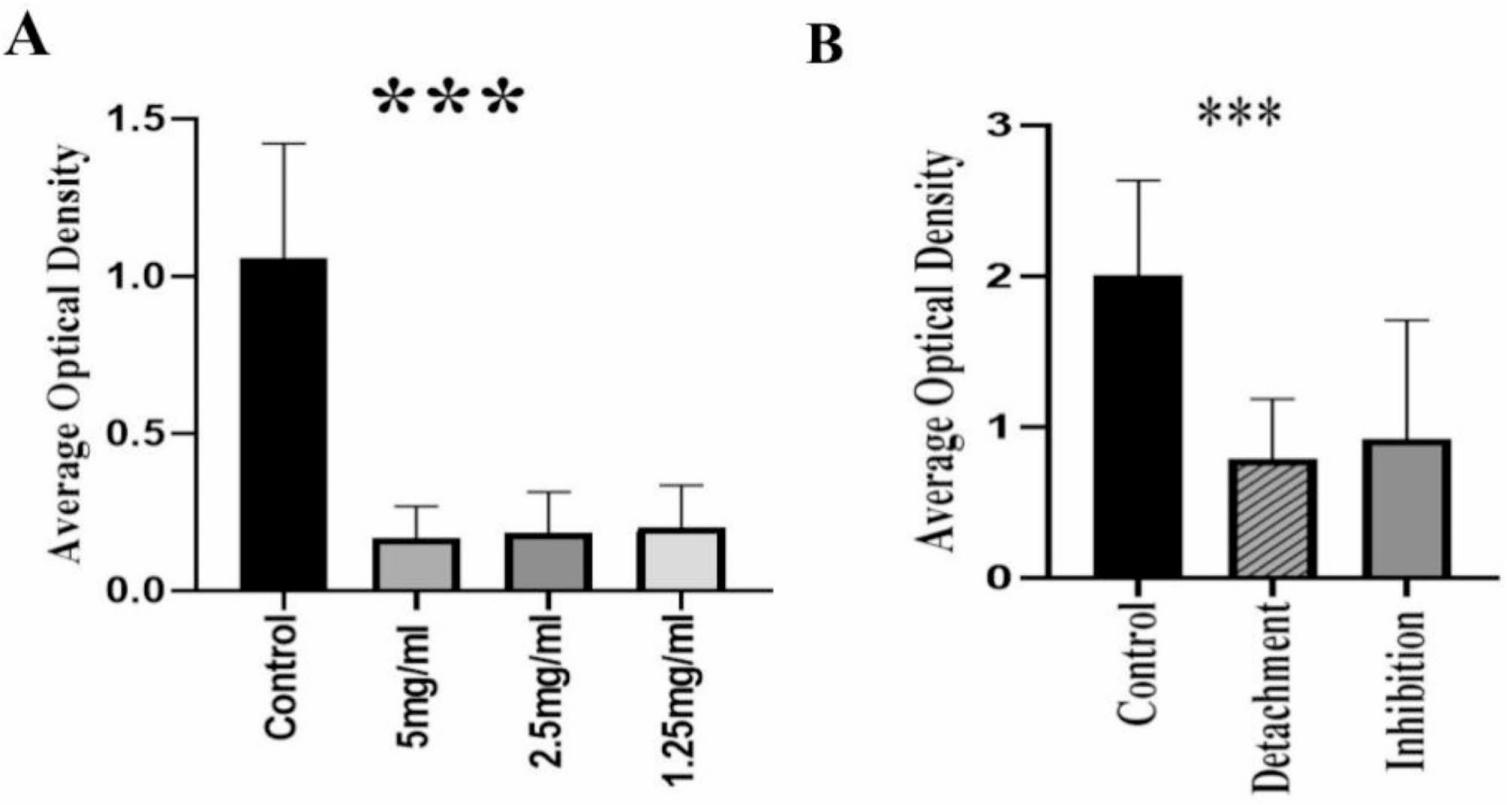
Effect of Pluronic F-127 on *P. aeruginosa* biofilm formation and detachment. The bar graphs represent the average optical density (OD) values measured using a crystal violet biofilm assay. (**A**) Biofilm formation in the presence of different concentrations (5 mg/mL, 2.5 mg/mL, and 1.25 mg/mL) of Pluronic F-127 compared to the untreated control (*p* < 0.001) (**B**) Comparison of biofilm optical density between untreated control, biofilm detachment, and biofilm inhibition conditions. Both detachment and inhibition groups exhibited a significant reduction in biofilm biomass relative to the control (*p* < 0.001)

Almost all biofilm forming isolates (*n* = 111) showed biofilm inhibition by Pluronic F-127 by 82%, 80% and 78% in concentration of 5 mg/mL, 2.5 mg/ml and 1.25 mg/ml, respectively. The lowest tested concentration of Pluronic F-127 (1.25 mg/ml) demonstrated significant biofilm inhibition especially on strong and moderate forming isolates (72%), 5 mg/ml of Pluronic F-127 showed a significant effect on strong biofilm producers with 82% inhibition. Regarding inhibition of adhered biofilms, results showed that 10 mg/ml of Pluronic F-127 break down the already established biofilm by 90%, most observed in strong biofilm forming isolates. A statistically significant difference was observed regarding the three concentrations of Pluronic F-127 in correlation to control (*P* < 0.05) suggesting Pluronic F-127 as one of the potent antibiofilm agents (Fig. 4).

### Scanning Electron microscope

**Fig. 5:**
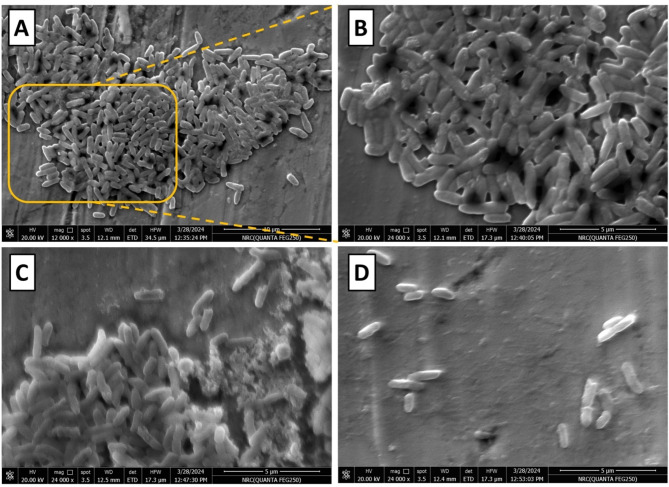
Scanning Electron Microscopy (SEM) images of *P. aeruginosa* biofilms (A) High-magnification SEM image showing a dense bacterial biofilm structure on a surface, the yellow box highlights a region of intense bacterial aggregation. (B) Closer view of the biofilm, showing bacterial cell morphology and dense clustering. (C) Partial biofilm disruption, revealing individual bacterial cells and extracellular matrix degradation (D) Significant biofilm reduction indicating strong inhibition effects

Biofilm inhibition by Pluronic F-127 was also confirmed by imaging using Scanning Electron Microscope. Untreated strains show a high percentage of exopolysaccharide matrix with increased aggregation of cells in thick multicellular pattern with an obvious coating layer representing the biofilm (Fig. 5: **A**, **B**). While cells treated with Pluronic F-127 showed cell aggregates with clear absence of biofilm coating (Fig. 5C) or isolated cells (Fig. 5D).

### Effect of pluronic F-127 on expression of biofilm genes

A slight change was observed regarding *lasR* and *rhlR* genes, with *ΔΔC*_*t*_ = 0.9836 for *rhl*R gene and *ΔΔCt* = 1.0176 for *lasR* gene which is still non-significant to be considered as a down regulation of QS-regulating genes.

## Discussion

The problem of antimicrobial resistance is well recognized worldwide, sending us back to the dark ages of the pre-antibiotic era. The healthcare sector in Egypt has been long suffering from this matter [42–44]. Consequently, the Ministry of Health and Population has lately issued the Egypt National Action Plan for Antimicrobial Resistance (2018–2022) based on the call of the World Health Organization (WHO) [45]. *Pseudomonas aeruginosa* is a highly infectious organism that poses a considerable barrier in healthcare settings, necessitating the rapid development of novel ways for its control and treatment. Biofilm formation plays a critical role in the antibiotic resistance of *Pseudomonas aeruginosa* by providing a physical barrier that limits antimicrobials penetration, altering metabolic activity of bacterial cells within the biofilm, and facilitating the persistence of dormant cells [46].

In the present study, 118 *P. aeruginosa* clinical isolates were isolated from samples collected from four different Egyptian hospitals, mostly from pus and urine samples. Several studies have also reported a high prevalence of *P. aeruginosa* isolates in pus and urine beside sputum [47–49]. All the isolates showed a high antimicrobial resistance profile representing MDR and XDR resistance patterns. Probably, the well-known overuse and misuse of antibiotics in the Egyptian hospitals stands behind such alarming profile, which calls for stringent prescription guidelines to address the issue. Similar patterns were reported earlier in some studies [44, 50, 51].

Surprisingly, among the 14 tested antibiotics in the present study, polymyxins (colistin and polymyxin B) were the most effective antibiotics. It is worth mentioning that generally polymyxins are kept as the last resort for combating bacterial infections to avoid the development of microbial resistance. Both antibiotics work through binding to the lipopolysaccharide and disrupt the outer and inner membranes of the Gram-negative bacteria [52]. These results were compatible with the retrospective study conducted in Saudi Arabia in 2020 and in Bangladesh in 2024 which showed high sensitivity of *P. aeruginosa* isolates to colistin [51, 53].

Carbapenems are also a potent group of antibiotics that are widely used in Egypt to treat infections caused by MDR *P. aeruginosa*. However, there is growing global concern about the emergence of carbapenem-resistance [54]. The high resistance rates of *P. aeruginosa* in our study agrees well with the reported pattern of the high prevalence carbapenemase-producing *P. aeruginosa* elsewhere [55]. *P. aeruginosa* harbors several genes encoding for beta-lactam degradation including genes encoding for metallo-β-lactamase (MBL) and *ampC* genes. These genes provide the bacteria with distinct pattern in resisting antimicrobial treatments [56]. The detection of *ampC* gene in 69% of the isolates revealed its association with ceftazidime resistance, in which over 90% of ceftazidime-resistant isolates harbored *ampC* gene. These results agreed with results of a study conducted in 2007, which investigated the prevalence of *ampC* over-expression as a mechanism of resistance accounted for β-lactam resistance in *P. aeruginosa* obtained from a hospital in Houston, TX, USA [57].

**Fig. 6:**
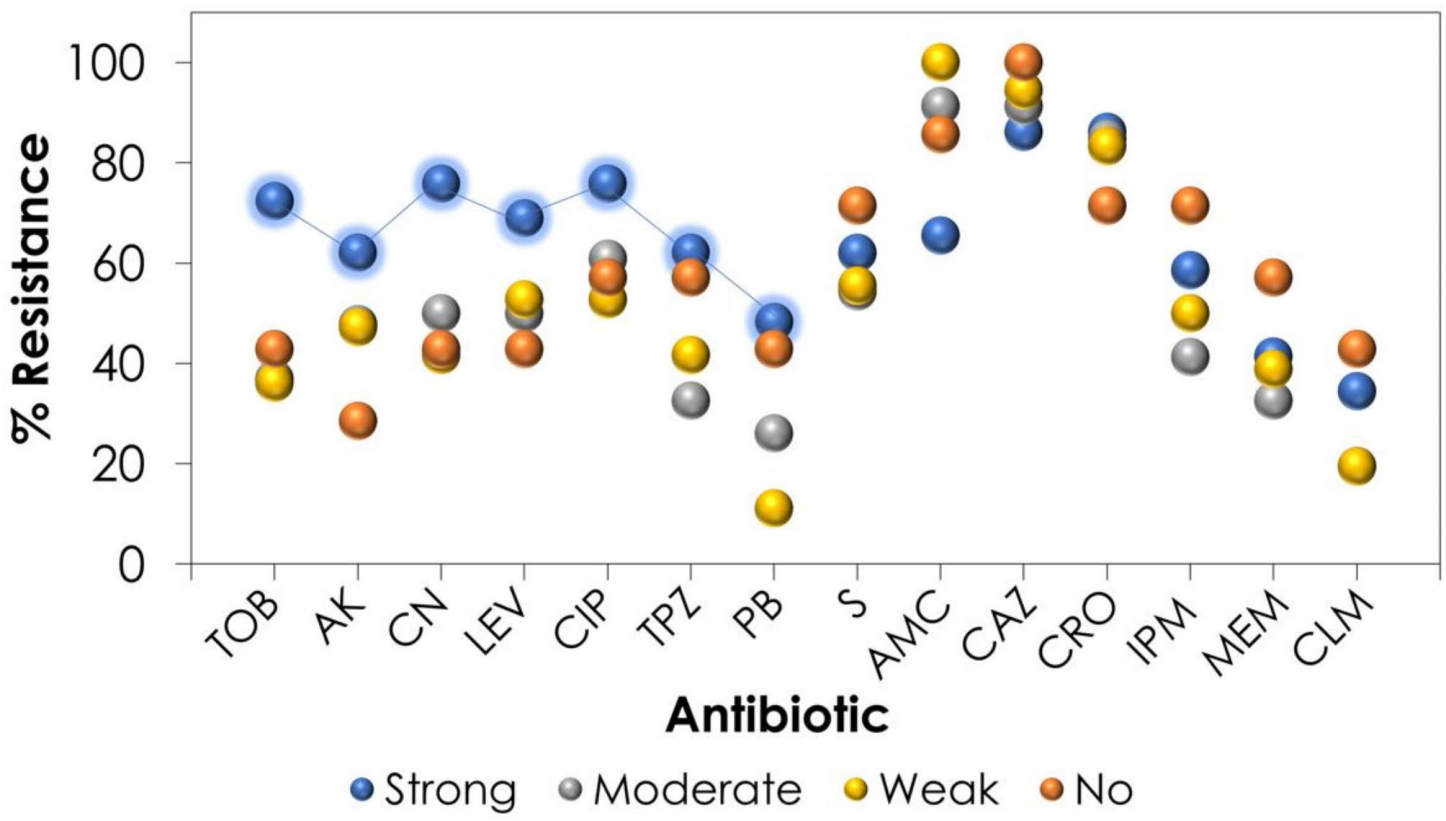
The percentage of resistance among each category of biofilm producers on *x* axis towards different antibiotics on *y*-axis showing the effect of biofilm formation on resistance pattern. Strong biofilm producers (blue), moderate biofilm producers (grey), weak biofilm producers (yellow), and non-biofilm producers (orange)

Interestingly, the strong biofilm formation ability of *P. aeruginosa* was linked to higher level resistance to certain antibiotics than moderate, weak and non-biofilm producers. These antibiotics were TOB, AK, CN, LEV, CIP, TPZ and PB, indicating the important role of biofilm formation in the development of antimicrobial resistance (Fig. 6). Moreover, the distribution of XDR isolates was more prevalent in strong biofilm producers (38% of all XDR isolates) followed by weak and moderate biofilm producers and finally non-biofilm producers (Fig. S2). Even after normalization with regard to the number of isolates in each category, around 44.8% of strong biofilm producers were XDR, compared to only 23.5% and 29.4% among moderate and weak biofilm producers, respectively.

In addition to antibiotic resistance, *P. aeruginosa* harbors a variety of constantly evolving extracellular virulence factors, among them QS-regulated virulence proteins assist the bacteria in escaping the host immune system. In this study, the majority of the isolates were biofilm-producers, which were further categorized to three levels: strong, moderate and weak. The results of biofilm formation agreed with that conducted by Sajid et al. (2019) who showed that 84% of *Pseudomonas* isolates have biofilm forming capability [58]. Swapna et al. (2022) mentioned a higher percentage up to 97% of strains were biofilm producer [47]. Asghari et al. (2021) stated that *P. aeruginosa* was dominant biofilm producers among other tested organisms and as the best biofilm-forming bacteria [59]. However, these results differs from those of Shrestha et al. (2019) and Kulkarni et al. (2020) who reported lower rates of biofilm production 32% and 26% among their isolates [48, 49]. Probably, the hospital hygiene and cleansing protocols might be responsible for generating variant with higher ability to form biofilm than others [60].

Beside the biofilm formation, microbial motility is another crucial pathogenic element of *P. aeruginosa*. Both play significant roles in bacterial pathogenicity, specifically in adhering to host cells [61]. Two different motilities were identified in *P. aeruginosa*, swarming and swimming. Results of swarming and swimming motilities were related to biofilm formation capability. Swimming was more prevalent among the isolates (92%) followed by swarming (77%). These findings agreed with study conducted in 2023 which confirmed that swimming motility is present in majority of the isolates followed by swarming ones [62]. Nassar et al. (2022) found that 100% undergoes both motility and a statistical positive significant correlation was observed between biofilm formation and motility of isolates which agreed with our findings [63]. Based on our results, we have observed a higher correlation between biofilm formation and swarming motility than with swimming motility, since most non-biofilm producers didn’t show swarming motility (71%). On the other hand, almost all non-biofilm producers exhibited positive swimming motility.

A strong correlation was observed between the presence of *las*R and *rhl*R genes, and biofilm formation. Results of *las*R gene as a main contributor in QS system in biofilm formation agreed with study conducted in Egypt which confirmed a significant correlation between *las*R-positive isolates and *las*R-negative isolates regarding biofilm production [34]. Mahmoud et al. (2021) revealed that there was highly significant relation between biofilm formation and detection of *las*R gene in which the *las*R gene was detected in 42 (77.8%) out of 54 *P. aeruginosa* isolates collected, while only 12 (22.2%) isolates did not harbor the gene [64]. In a similar manner, there was a strong correlation between the presence of *rhl*R gene and biofilm production. Results agreed with results of Ghanem et al. (2023) which showed high prevalence of *rhl*R gene in MDR isolates (94%) [53]. Our study was coincident with that of Mohamed et al. (2023) where *las*R and *rhl*R genes were detected in 89% and 82%, respectively, of biofilm forming isolates from ocular infections, and confirmed that there was a statistically significant difference between both genes and biofilm forming capability [65]. Results of *rhl*R gene conflict with El Negery et al. (2021) which showed that there was no statistically significant difference between *rhl*R-positive isolates and *rhl*R-negative isolates regarding biofilm production [34]. On the other hand, a study conducted in Egypt revealed that *las*R and *rhl*R genes were identified in all *P. aeruginosa* isolates [66]. Results of *las*R and *rhl*R genes in correlation with motility assay revealed a strong association between presence of both gene in biofilm forming producing isolates with presence of swarming or swimming motility, negative *las*R and *rhl*R genes and no motility pattern appeared in strains lack ability to form biofilm and in only three weak biofilm forming strains. These findings confirmed a previous study which indicated that *las* and *rhl* systems are required for twitching motility in *P. aeruginosa* [67].

Another virulence factor expressed by *P. aeruginosa* is endotoxin A, which is responsible for the cytotoxic capacity and stimulation of pro-inflammatory cytokine synthesis. The encoding gene, *toxA*, was detected in 95% of isolates and 100% in XDR ones. This result agreed with results conducted in 2023, in which 100% of isolates expressed *toxA* gene [68]. Results also agreed with Sabharwal et al. (2014) in which all MDR isolates were positive for *toxA* gene [69].

**Fig. 7:**
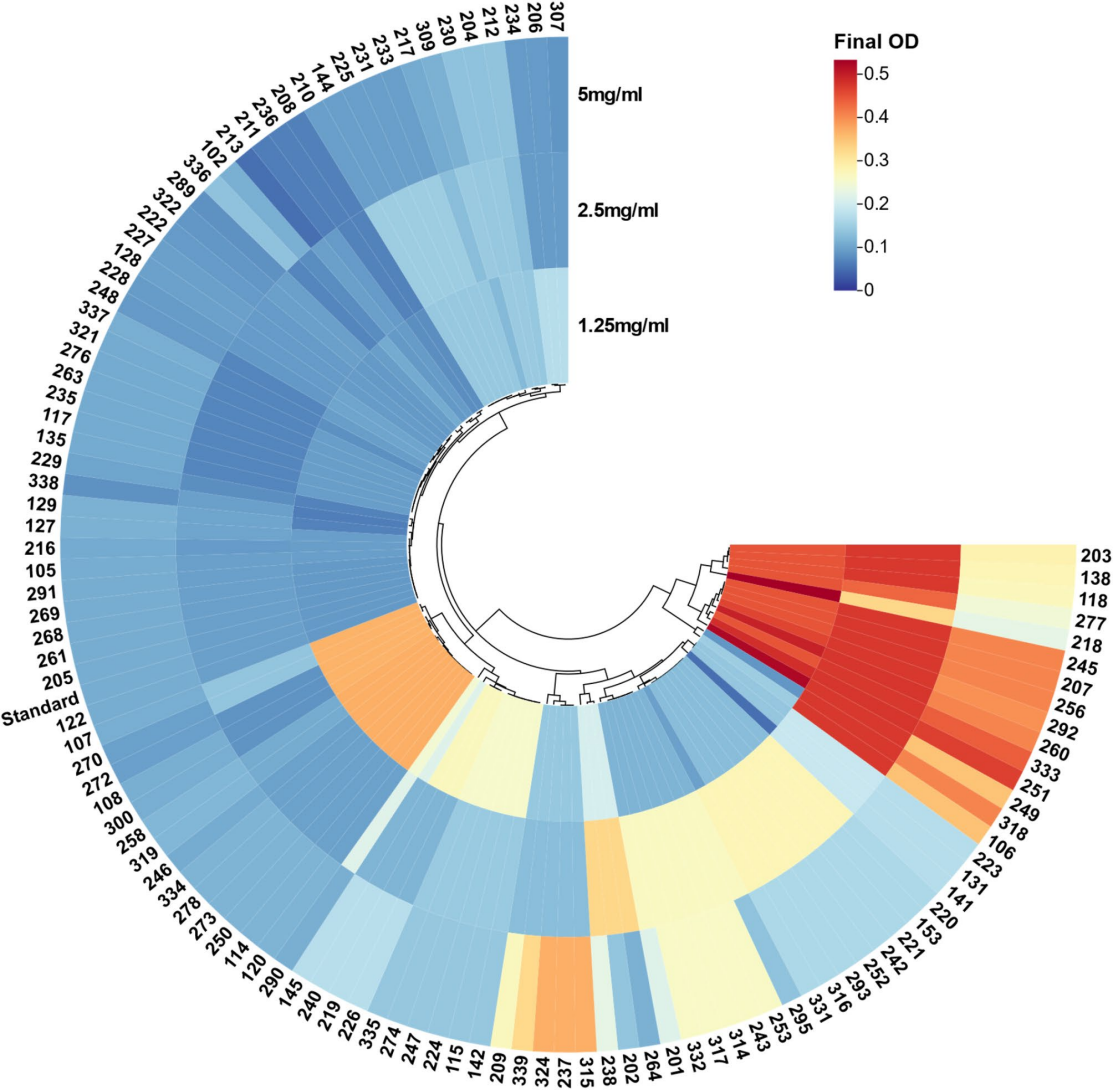
Heatmap showing the effect of Pluronic F-127 (1.25, 2.5 and 5 mg/ml) on biofilm formation by clinical *P. aeruginosa* isolates represented as final OD measured at 595 nm using the crystal violet technique. The image was generated by ChiPlot (Chiplot.online)

To overcome the biofilm formation, Pluronic F-127 was evaluated as a potential candidate. This strategy is promising since it acts on the biofilms through a non-biocidal method, in contrast to antibiotics and biocides, which can be harmful and encourage the emergence of resistance [70]. Pluronic F-127 showed a significant effect on *P. aeruginosa* biofilm formation (Fig. 7). The average OD without Pluronic F-127 treatment was 1.06 ±0.36 and was reduced to 0.17 ±0.1, 0.18 ±0.13 and 0.2 ±0.13 with 5, 2.5 and 1.25 mg/ml Pluronic F-127, respectively. These results agreed with study conducted in 2020 evaluating Pluronic F-127 on different bacterial and fungal biofilm-forming species including *P. aeruginosa* which revealed that 80% inhibition of biofilm [16]. Another study investigates the effect of Pluronic F-127 on *P. aeruginosa* clinical strains showing no inhibition on biofilm formed, while showed a significant inhibition of biofilm by 90% in *Staphylococcus epidermidis* clinical isolates. The study concluded that Pluronic F-127 has no activity on *P. aeruginosa* as a result of cell surface hydrophobicity in comparison to the Gram-positive *S. epidermidis* which disagree with our findings [38]. Alvarado-Gomez et al. (2018) investigated the effect of Pluronic F-127 on both *P. aeruginosa* and *S. aureus*, a significant effect was observed on both pathogen [71].

Although Pluronic F-127 had a considerable activity towards most of the isolates (∼85% reduction in the OD_595_ compared to the control without Pluronic F-127), some isolates (∼10% of the isolates) were affected to a lower extent (∼50% reduction in the OD_595_ compared to the control) than the others this is may be as a result of the resistance pattern of these strains;60% of these isolates were MDR and 40% were XRD. Moreover, almost half of these isolates were collected from urine catheter with high tendency to form biofilm and have been long exposed to antibiotics secreted in the urine. Therefore, we believe that the eradication of the biofilm can be quite challenging for these strains. In addition to this, these strains showed both positive swarming and positive swimming motility which may contribute to this observation. On the other hand, Pluronic F-127 showed better effect on detachment of pre-formed biofilms by these isolates. These isolates are clustered at the end of the heatmap shown in Fig. 7 in red. The assay also revealed that Pluronic F-127 has good activity even at the lowest tested concentrations (1.25 and 2.5 mg/ml).

Despite Pluronic F-127’s effectiveness in disrupting biofilms, some bacterial isolates show reduced susceptibility due to several factors. Genetic variation among strains can lead to adaptations that enhance resistance to Pluronic F-127, with specific gene variations affecting adhesion and EPS production [72]. The age and maturity of biofilms influence susceptibility; mature biofilms often have a dense matrix that provides protection against antimicrobial agents, making them less affected by Pluronic F-127 compared to newer ones [73]. Furthermore, external conditions such as nutrient availability, pH, and temperature can also impact the effectiveness of Pluronic F-127 on these isolates [74].

Pluronic F-127 was also evaluated for the ability to detach the pre-formed biofilm at a concentration of 10 mg/ml. The test revealed that about 90% of pre-formed biofilm was successfully detached indicating that Pluronic F-127 acts well on pre-formed biofilm. Real time expression of *las*R and *rhl*R genes after treatment with Pluronic F-127 revealed a non-significant change in expression levels of both genes. This is due to the unclarity of the mechanism of Pluronic F-127 in inhibiting biofilm and the wide range of QS genes associated in biofilm formation. In our opinion this may be due to the better effect of Pluronic F-127 on the detachment of biofilm after establishment than on biofilm inhibition.

## Conclusion

*Pseudomonas aeruginosa* infections pose a significant health concern. The ability of bacteria to form biofilms contributes to the development of antibiotic resistance, which in turn leads to the occurrence of persistent and chronic bacterial illnesses. Many isolates exhibited moderate to strong biofilm forming ability. Although discovering a new potent antibiotic for treating highly resistant organisms is important, a promising alternative strategy is to focus on inhibiting transmission. Pluronic F-127 is a secure and biocompatible substance which is utilized in multiple medical products. The results demonstrated a promising level of biofilm inhibition and detachment in most isolates. It has a chance to serve as a substitute means for combating biofilm formation. Our findings provide significant insights based on in-vitro models; the absence of in-vivo ones limits the direct applicability of the results to clinical scenarios. We recommend the need for future studies to validate these findings in appropriate animal models.

## Electronic supplementary material

Below is the link to the electronic supplementary material.

Additional file 1: **Table S1**: Demographics of collected strains, **Figure S1**: Agarose gel images showing the prevalence of *toxA*, *ampC*, *Las*R, and *rhl*R genes, and **Figure S2**: The distribution of XDR isolates in relation to biofilm forming capacity

## Data Availability

The data used and/or analyzed during the current study are available from the corresponding author (Mai Hamed Salem) on reasonable request.

## Abbreviations

CDC: Center for Disease Control and Prevention
CF: Cystic fibrosis
CLSI: Clinical and Laboratory Standards Institute
COPD: Chronic obstructive pulmonary disease
CV: Crystal violet
EPS: Exopolysaccharide matrix
MBL: Metallo-β-lactamase
MDR: Multi-drug resistant
MIC: Minimum Inhibitory Concentration
MTPM: Microtiter plate method
OD: Optical density
*P. aeruginosa*: *Pseudomonas aeruginosa*
PCR: Polymerase chain reaction
PDR: Pan-drug resistant
PEO: Poly- (ethylene oxide)
PPO: Poly- (propylene oxide)
PQS: Pseudomonas quinolone signal
QS: Quorum sensing
SEM: Scanning Electron Microscope
TAE: Tris-Acetate EDTA
TSB: Trypticase soya broth
VAP: Ventilator-associated pneumonia
XDR: Extensive drug resistant
