# Supplementary material for: Pseudomonas aeruginosa clinical isolates in Egypt: phenotypic, genotypic, and antibiofilm assessment of Pluronic F-127
Source: BMC Microbiol. 2025 Apr 25;25:245. doi: 10.1186/s12866-025-03946-0 (PMC12023487; doi:10.1186/s12866-025-03946-0)
Supplement: Supplementary file 1 — Additional file 1: Table S1: Demographics of collected strains, Figure S1: Agarose gel images showing the prevalence of toxA, ampC, LasR, and rhlR genes, and Figure S2: The distribution of XDR isolates in relation to biofilm forming capacity [file 12866_2025_3946_MOESM1_ESM.docx]

**Electronic Supplementary Material**

***Pseudomonas aeruginosa* clinical isolates in Egypt: phenotypic, genotypic, and antibiofilm assessment of Pluronic F-127**

*Mai Hamed Salem^1,2^*, Ahmed F. Azmy^2^, Tarek Dishisha^2^, Nesrein Dessouky^1^*

*^1^Department of Microbiology and Immunology, College of Pharmaceutical Sciences and Drug Manufacturing, Misr University for Sciences and Technology, P.O. Box 77, Giza, Egypt*

*^2^Department of Pharmaceutical Microbiology and Immunology, Faculty of Pharmacy, Beni-Suef University, 62511 Beni-Suef, Egypt*

* Corresponding authors:

**E-mail address:** may.hamed@must.edu.eg

**Tel:** +201124776488

**Table S1. Demographics of collected strains**

| **Demographics** | | ***P. aeruginosa* (n=118)** | **Percentage** |
| --- | --- | --- | --- |
| **Gender** | Male | 69 | 58.5% |
|  | Female | 49 | 41.5% |
| **Age Group** | <1-20 | 40 | 34% |
|  | 21-40 | 16 | 13.55% |
|  | 41-60 | 55 | 46.6% |
|  | 61-80 | 7 | 5.9% |
| **Specimen** | Pus | 37 | 31.36% |
|  | Urine | 24 | 20.34% |
|  | Wound | 15 | 12.71% |
|  | Endotracheal tube | 14 | 11.86% |
|  | Blood | 13 | 11.02% |
|  | Sputum | 10 | 8.47% |
|  | Vaginal swap | 5 | 4.24% |

**Figure S1**

**
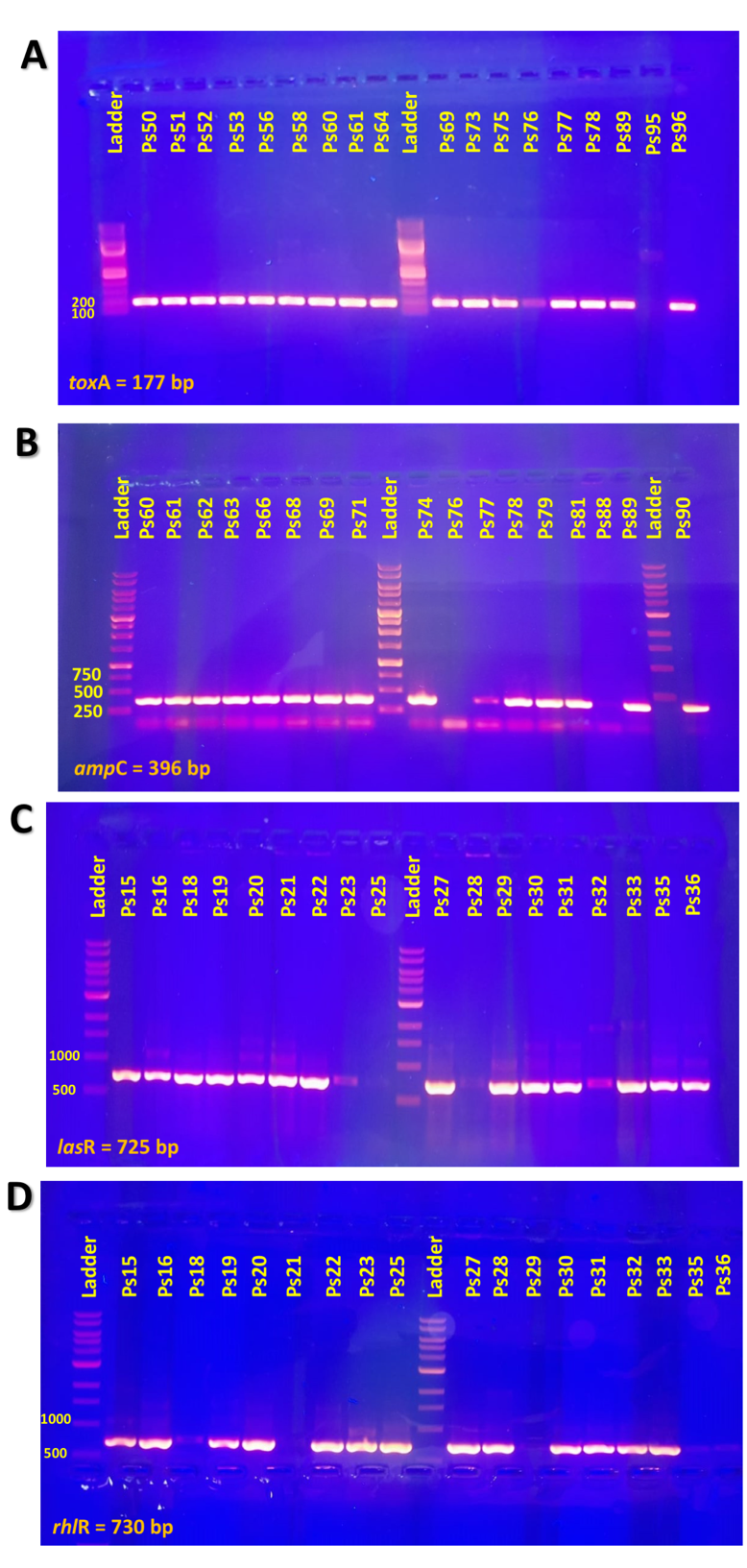
**

**Fig. S1.** A: *toxA* gene appeared at 177 bp using 100 bp DNA ladder all isolates were positive for *toxA* gene , B: *ampC* gene appeared in 396bp, all isolates were *ampC*+ except for Ps76 and Ps88 isolates, C: *las*R gene appeared in 725bp using 1Kb ladder; all isolates were positive for *las*R except for Ps25 isolate, D: *rhl*R gene appeared in 730bp whereas Ps21,Ps29,Ps35and Ps36 were negative for *rhl*R.**Figure S2**


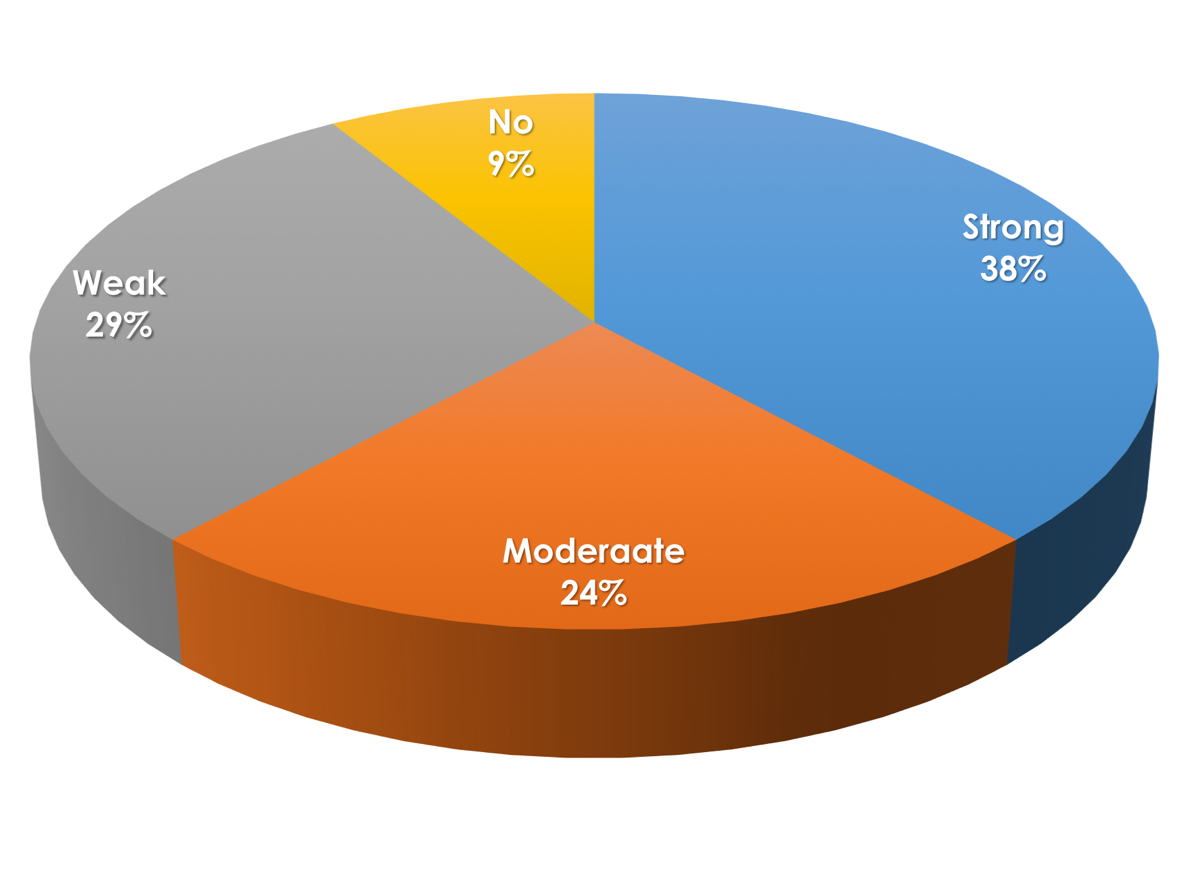


**Figure S2.** The distribution of XDR *Pseudomonas aeruginosa* isolates in relation to biofilm forming capacity.
